# Clarifying life lost due to cold and heat: a new approach using annual time series

**DOI:** 10.1136/bmjopen-2014-005640

**Published:** 2015-04-15

**Authors:** Nirandeep Rehill, Ben Armstrong, Paul Wilkinson

**Affiliations:** 1 London Kent Surrey & Sussex Public Health Training Programme, London, UK; 2 Department of Social and Environmental Health Research, Faculty of Public Health and Policy, London School of Hygiene & Tropical Medicine, London, UK

**Keywords:** Aged, Climate Change, Humans, London, Mortality, Temperature

## Abstract

**Objective:**

To clarify whether deaths associated with hot and cold days are among the frail who would have died anyway in the next few weeks or months.

**Design:**

Time series regression analysis of annual deaths in relation to annual summaries of cold and heat.

**Setting:**

London, UK.

**Participants:**

3 530 280 deaths from all natural causes among London residents between October 1949 and September 2006.

**Main outcome measures:**

Change in annual risk of death (all natural cause, cardiovascular and respiratory) associated with each additional 1°C of average cold (or heat) below (above) the threshold (18°C) across each year.

**Results:**

Cold years were associated with increased deaths from all causes. For each additional 1° of cold across the year, all-cause mortality increased by 2.3% (95% CI 0.7% to 3.8%), after adjustment for influenza and secular trends. The estimated association between hot years and all-cause mortality was very imprecise and thus inconclusive (effect estimate 1.7%, −2.9% to 6.5%). These estimates were broadly robust to changes in the way temperature and trend were modelled. Estimated risk increments using weekly data but otherwise comparable were cold: 2.0% (2.0% to 2.1%) and heat: 3.9% (3.4% to 3.8%).

**Conclusions:**

In this London annual series, we saw an association of cold with mortality which was broadly similar in magnitude to that found in published daily studies and our own weekly analysis, suggesting that most deaths due to cold were among individuals who would not have died in the next 6 months. The estimated association with heat was imprecise, with the CI including magnitudes found in daily studies but also including zero.

**Strengths and limitations of this study**

- This study uses data on mortality and temperature collected over six decades in London to find that colder years were associated with increased mortality, with 2.3% (95% CI 0.7% to 3.8%) more deaths occurring per degree of cold during the year.
- The study provides estimates of the effect of temperature on mortality which are resistant to short-term harvesting (deaths brought forward by up to 6 months).
- Like all epidemiological studies, there is the potential for residual confounding by uncontrolled risk factors.
- Estimates of annual deaths in relation to heat were too imprecise to draw conclusions about life shortening in excess deaths due to heat.

## Introduction

There is ample evidence that mortality increases on and immediately after hot or cold days.^1–3^ This is often cited to make the case for public health action.^4^ ^5^ However, there is limited information on the duration of life lost by these excess deaths. It is possible that the excess deaths might be wholly or partly among very frail people who are approaching the end of their life and cause very little shortening of life.^6^ This phenomenon is commonly termed *harvesting*, *mortality displacement* or *deaths brought forward*, and some evidence exists to support it, particularly with respect to heat-related deaths.^7–9^ The effect of temperature on mortality is greatest among the elderly,^1^ ^10^ ^11^ and may also be more common among those with existing comorbidities.^1^ However, the extent to which harvesting occurs is debated.

Some daily studies have used distributed lag and related approaches to estimate very short-term harvesting (roughly 1 month or less) by looking for mortality decreases in the weeks after hot spells. Estimates of harvesting from such studies range from 70% of all heat-associated deaths in 15 European cities,^12^ to fewer than 10% of deaths during the 2003 heatwave in France.^13^ Less evidence of short-term mortality displacement exists for cold-associated deaths, possibly because the longer lag associated with cold effects makes the distributed lag approach to estimating harvesting problematic.^7^ Also, it is very difficult with daily studies to identify harvesting beyond a month or so.^14^ This would require identifying mortality deficits a few months down the line, which is difficult because of noise and the need to control for seasonal and other temporal variation not due to temperature.

Some studies have demonstrated that higher-than-average overall deaths in winter lead to lower daily temperature effects on mortality the following summer.^15^ ^16^ A cohort study found that years of high summer temperature variability were associated with high mortality.^17^ These studies bring indirect evidence that some temperature-related deaths were displaced by at least several months, but not directly on the extent of displacement due to heat and cold.

The Excess Winter Deaths Index approach used by the UK's Office for National Statistics identifies excess mortality within geographical regions over the 4 months of ‘winter’ (defined as December to March), compared with the preceding 4 and following 4 months. This is reasonably resistant to short-term displacement but could be biased by displacement from winter to non-winter in the same year. Some of these deaths, furthermore, may be attributable to seasonal respiratory infections such as influenza, rather than temperature per se.

Given the limited evidence on the extent of mortality displacement due to heat and cold from previous studies, this study set out to avoid some of the limitations of current techniques by applying time series methods to investigate whether annual death counts have been associated with annual summaries of temperature extremes. If so, it would suggest that those deaths would not have otherwise occurred in that year. Thus, the analysis will provide evidence on the extent to which temperature-associated deaths represent only short-term displacement.

## Methods

### Mortality data

We examined deaths among London residents from all natural causes between October 1949 and September 2006, using two sources of mortality data to cover the whole study period. For 1949–1975, we used digitised weekly counts of death registrations published in print by the Registrar General,^18^ supplementing 1950–1964 data from a previous study.^19^ For 1976–2006, we used daily counts of death collated for other studies^20^ ^21^ and originally obtained from the Office of National Statistics (ONS). The data were for London Administrative County for 1949–1965, and for the larger Greater London thereafter. For all years, we retrieved deaths due to all natural causes, cardiovascular causes and respiratory causes. The daily counts were collapsed into weeks to create a complete series of weekly mortality for the entire 57 years of study, starting from 2 October 1949.

For the primary analyses, we aggregated the weekly data into years starting in autumn (first week of October), rather than conventional calendar years. This was to minimise exposure measurement bias, given that deaths due to cold can be delayed by as long as 3 weeks^8^; thus, deaths in January could be the result of cold weather during the preceding December. Heat-related mortality effects are predominantly seen within 1–3 days,^1^ so it is unlikely that October would include deaths due to hot weather in September. Weeks were numbered sequentially from the start date and organised into years of 52 or 53 weeks, with each year starting in early October. All weeks numbered as the 53rd week in that year were dropped from the series to leave summed counts of deaths for fifty-seven 52-week years, for simplicity in the regression model.

### Boundary and ICD changes

The change in the administrative definition of London in 1966 lead to a sharp artificial jump in death counts. Because the years in our study started in October, this led to two jumps in annual death counts, which we modelled using two indicator variables to make ‘steps’ going into 1965–1966 and 1966–1967. Other smaller discontinuities considered in sensitivity analyses were similarly allowed for as were those for revisions of the International Classification of Diseases (ICD; affecting only cause-specific analyses) for which no bridging code was available. Details are provided in online supplementary appendix A.

### Meteorological data

We downloaded daily temperatures recorded at Heathrow airport (the only station with data available for the whole period) from the British Atmospheric Data Centre (BADC). In order to capture heat or cold exposures in one annual summary statistic, we first identified daily measures of ‘heat’ and ‘cold’ by assuming the V-shaped model often used in daily studies. To inform choice of the minimum mortality temperature (apex of the V; common cold and heat threshold), we reviewed 11 previous studies examining temperature and mortality in London (see online supplementary appendix B). There was no clear consensus over studies, probably due to differences in methods (lags, shape of model). For our primary analysis, we chose a value of 18°C of daily mean temperature as a common threshold both for cold and heat as it approximated the mean over the reviewed studies. Because of its uncertainty, the choice of threshold was prioritised for exploration using sensitivity analyses.

‘Heat-degrees’ was derived for each day as the number of degrees *above* 18°C of the daily mean temperature, while cold-degrees was defined as the number of degrees *below* 18°C of the daily mean temperature. Annual means of these measures, ‘annual-heat’ and annual-cold were used in our analyses.

### Statistical analysis

We carried out a quasi-Poisson time series regression analysis, with yearly all-cause natural deaths as the outcome, and the main exposures of interest being annual-heat and annual-cold. We undertook a primary analysis based on a model informed by a priori judgement but explored sensitivity to assumptions in that model.

We used indicator variables to account for steps in the mortality series in 1965–1966 and 1966–1967 due to a boundary change (discussed above). In the cause-specific analyses, we also included four further indicator variables to reflect steps anticipated due to ICD changes. To control for long-term trend in the model, we included a natural cubic spline function assuming 1 degree of freedom for every 10 years of data (5 degrees of freedom in total), equating to a roughly 10-year moving average. We chose this degree of flexibility by judgement to allow control for gradual changes in population size, age structure and death rates, while leaving enough variability to use in analyses.

We adjusted for influenza epidemics by including as an explanatory variable the proportion of deaths each year that were classified as caused by influenza.

Details of the main model are provided in online supplementary appendix C. Alternative model assumptions, including different degrees of confounder control, were considered in sensitivity analyses.

For comparison with the annual time series estimates, we also undertook a simple time series regression of weekly counts following conventional methods, using the same heat-degrees and cold-degrees daily measures aggregated to weeks (‘week-heat’ and ‘week-cold’). We controlled confounding by seasonal and other time-varying risk factors by stratifying by year and month (344 strata), using a conditional quasi-Poisson model (equivalent to a time-stratified case-crossover). Because of the known lag between cold and mortality excess, the cold variable included in the model was the mean of the daily cold-degrees over that week and the previous one.

All analyses were performed in Stata V.11.2. The annual data set and core code are available from the Dryad Digital Repository: http://dx.doi.org/10.5061/dryad.02k83.^22^

## Results

**Figure 1:**
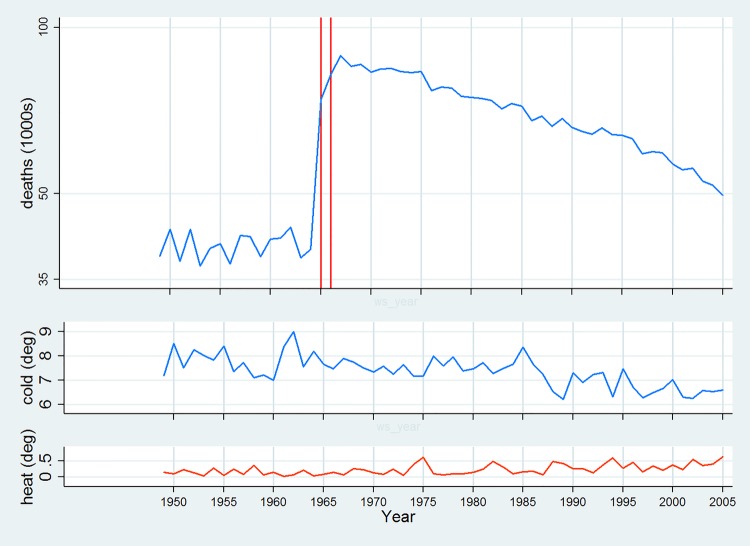
Annual deaths and mean of daily degrees Celsius below/above 18°C, London 1949–2006 (vertical lines indicate years affected by boundary changes). Points and lines are graphed at the first year of the October–September years used in analyses, for example, analysis year October 1965 to September 1966 is ‘1965’.

Our data set comprised 57 annual counts totalling 3 530 280 deaths from natural causes for years from 1949–1950 to 2005–2006. Over the entire period, apart from a sharp increase due to the changed administrative definition of London in January 1966, there is evidence of a gradual decline from about 1970 (figure 1).

Mean daily temperature exceeded the threshold of 18°C on 11.1% of days, and was below this threshold on 88.7% of days. For each year during the study period, the mean cold-degrees over the year (degrees below 18°C) was on average 7.4°C and varied between 6.2°C and 9.0°C (figure 1). Mean heat-degrees (degrees above 18°C) was much lower (0.2°C), and varied only from 0°C to 0.6°C.

Table 1 presents the estimated increase in mortality for each degree of cold and heat across the year, as determined by the regression model. Overall, cold years were associated with increased deaths from all causes. For each additional degree of cold across the year, all-cause mortality increased by 2.3% (95% CI 0.7% to 3.8%), after adjustment for influenza and secular trend. The effect of cold was greater in those aged 65 and above, but CIs were wide. Colder years were also associated with proportionally more deaths from cardiovascular disease (2.9% per degree) and respiratory disease (7.6% per degree), but again CIs were wide.

**Table 1:** Per cent increase in annual deaths per degree Celsius of cold (daily average of degrees below 18°C) and of heat (daily average of degrees above 18°C)

	Per cent increase in mortality per °C (95% CI)	
	Main (annual data)	Comparative (week data)
*Cold*		
All causes		
All	2.3 (0.7 to 3.8)	2.0 (2.0 to 2.1)
Age 65+	2.4 (0.6 to 4.3)	2.2 (2.1 to 2.3)
Under 65	1.9 (−0.0 to 3.9)	1.5 (1.3 to 1.6)
Cardiovascular		
All	2.9 (0.9 to 5.0)	2.4 (2.3 to 2.5)
Respiratory		
All	7.6 (2.7 to 12.8)	3.6 (3.4 to 3.8)
*Heat*		
All causes		
All	1.7 (−2.9 to 6.5)	3.9 (3.6 to 4.1)
Age 65+	1.0 (−4.4 to 6.8)	4.5 (4.2 to 4.8)
Under 65	3.0 (−3.2 to 9.7)	2.0 (1.5 to 2.5)
Cardiovascular		
All	−0.1 (−5.9 to 6.1)	3.9 (3.5 to 4.3)
Respiratory		
All	3.3 (−10.3 to 19.0)	7.9 (7.2 to 8.6)

The estimated increase in mortality with heat (1.7% per degree) was very imprecise (−2.9% to 6.5%). Age-specific and cause-specific heat-related mortality were similarly imprecise, making comparisons unreliable.

A range of sensitivity analyses (table 2) showed that only where the spline used to allow for mortality trends over time was very inflexible (3 knots) did the association of mortality with cold change substantially (losing significance). This model, however, fit the data appreciably less well by Akaike's information criterion. The association of heat with mortality was more sensitive to model choices, but estimates of mortality increment due to heat remained very imprecise in all models. Residual analysis of the main model did not suggest problems of poor model fit (details in online supplementary appendix D).

**Table 2:** Sensitivity analyses

	Mortality increment (%) per degree below/above 18°C (95% CI)		
	Cold	Heat	AIC
Main model	2.3 (0.7 to 3.8)	1.7 (−2.9 to 6.5)	1943.7
Alternative distributional assumptions (main=overdispersed Poisson)			
Simple regression	2.1 (0.5 to 3.7)	1.8 (−2.5 to 6.4)	NA
Negative binomial	2.5 (1.1 to 3.8)	1.5 (−2.9 to 6.2)	NA
Alternative time spline (main=6)			
3-knot spline	1.1 (−0.9 to 3.1)	−0.2 (−6.2 to 6.2)	3043.8
9-knot spline	2.3 (0.8 to 3.9)	2.8 (−1.9 to 7.6)	1824.0
Additional step for smaller discontinuities (see web appendix A)			
Years starting 57 and 58	2.6 (1.0 to 4.2)	2.4 (−2.4 to 7.4)	1903.8
Year starting 64	2.4 (0.8 to 3.9)	1.6 (−3.0 to 6.4)	1909.5
Years starting 75 and 76	2.4 (0.9 to 3.9)	2.2 (−2.9 to 7.4)	1812.6
Alternative heat/cold threshold (main=18)			
Threshold 15°C	2.2 (0.5 to 4.0)	−0.2 (−2.8 to 2.4)	1966.5
Threshold 21°C	2.1 (0.8 to 3.5)	8.4 (−4.8 to 23.4)	1938.0
Influenza control			
No adjustment for influenza	3.0 (0.9 to 5.2)	6.3 (−0.2 to 13.2)	3152.1

AIC, Akaike's information criterion: (−2×ln(likelihood)+2(terms)); NA, not available.

The comparative conventional month-stratified time series analyses on weekly data for the same period gave all-cause estimated cold and heat effects as 2.0% (2.0% to 2.1%) and 3.9% (3.6% to 4.1%) per degree below and above, respectively. CIs were much narrower than for the annual data estimates, and point estimates for age-specific and cause-specific deaths showed clear pattern of higher relative risks in the elderly and in deaths due to cardiovascular and especially respiratory diseases.

## Discussion

In this annual time series, we found that over six decades in London colder years were associated with increased mortality, with 2.3% (95% CI 0.7% to 3.8%) more deaths occurring per degree of cold during the year. This suggests that cold-related deaths were brought forward by at least half a year. Cold weather in the UK typically occurs between December and March; had the excess of daily deaths associated with preceding weeks of cold weather been ‘due to occur’ within the same year as defined in this study—that is, before the following October—no association between cold and mortality would have been detected. Our findings were generally robust to variations in the modelling assumptions and regression techniques used.

There have, to our knowledge, been no other studies looking at long-term mortality in relation to long-term temperature. The observation, in a cohort study, that years of high summer temperature variability were associated with high mortality also adds evidence that temperature-related deaths were displaced beyond the year end.^17^ It is relevant, however, to compare our estimate with those from published daily studies, which show acute impacts of cold without excluding those displaced only by a few months. Two London studies used the same cold threshold as we did (18°C).^9^ ^23^ If all the excess deaths identified in the two daily studies have been displaced beyond the next October and there were no longer lag effects, the same increments in risk should appear in the daily as well as annual studies, setting aside the slightly different time periods of the studies (1988–1992 and 1993–1996). The daily studies both found increments of daily mortality of 1.4% per degree below 18°C.

We found similar estimates of increment in risk per degree of cold in our subsidiary weekly analysis (2.0%). That our estimate from annual data was positive and similar to our own weekly and other's daily estimates strongly suggests that the excesses in the daily and weekly studies were indeed displaced beyond October. Beyond that, that the annually based estimate is somewhat larger gives some suggestion that there may be adverse mortality effects operating at much longer lags than were detectable in the daily studies (lags 3 days and 2 weeks). The difference could however be due to chance, as 1.4% and 2% falls within the wide CI for this study (0.7% to 3.8%).

Estimates for the impact of heat from this study were very imprecise. In particular, the CI for increase in mortality per degree above 18°C, −2.9% to 6.5%, includes all the estimates made in daily studies, including the 1.3% found in the one daily study using the same 18°C threshold,^9^ and also the estimate presented in the current paper from weekly data (3.9%). This limit in precision precludes making any conclusion as to the presence or absence of short-term harvesting for heat-related deaths from this analysis. The precision limitation is due to the limited number and variation of hot days annually in London. Annual studies in cities with more days above their heat thresholds would likely have more power.

### Limitations

Like all epidemiological studies, this one was subject to residual confounding by uncontrolled risk factors. Many factors, including the size and demographic structure of the London population as well as declining age-specific death rates due to changes in healthcare and risk factors (eg, smoking), would have contributed to changes in counts of annual deaths. In common with other time series regression studies,^24^ we relied mainly on a smooth (spline) function of time to control for these, under the assumption that they would cause smooth changes. Although this cannot rule out residual confounding, it is reassuring that increasing the flexibility of the spline or adding further ‘step’ functions changed the estimated cold effect very little. The much lower estimate for cold when using a less flexible spline (table 2) has little credibility, given the much poorer fit of this model (Akaike's information criterion).

How and whether to control for influenza is problematic, as it is possible that it might be on the causal pathway between cold and death. Our approach, using the proportion of total deaths due to influenza as covariate in the regression, is thus we believe conservative.

Air pollution is an established risk factor and also changes over time. We were unable to control for this due to not having data for the complete period. However, data on black smoke for the period 1976–2003 were available,^25^ and analysis revealed a weak negative correlation between mean annual cold and black smoke during this time, after accounting for year (r=−0.25). This suggests that confounding from air pollution is unlikely to have been substantial, and control for it would be more likely to increase estimates of cold effects.

Despite the large population of London and the long duration of our series, power and precision was limited for some analyses. Heat effects we discussed above, and also the age-specific and cause-specific analyses of cold were too imprecise to allow much interpretation, even if point estimates followed broadly the expected pattern of higher risk in the elderly and for cardiovascular and especially respiratory deaths.^1^ ^26^ ^27^ The more precisely estimated patterns we found in the weekly analysis match those found by others.

Finally, our approach to summarising cold and heat in each year is not the only possible one. We chose it so as to maximise the link between this study and daily studies. However, in fact the annual measure of cold adopted (degrees below 18) was very highly negatively correlated, in temperate London, to annual mean temperature (r=−0.97). The heat measure was also correlated but less strongly so (r=0.81). Thus, analyses using mean annual temperature would have resulted in very similar estimates of cold effects and broadly similar heat effects.

### Public health implications

Given the imprecision of our estimate of 2.3% increased mortality per degree of cold (CI 0.7% to 3.8%), we do not translate it to a quantitative burden. We would in any case not expect the numerical estimate to be generalisable to other places. The importance of our finding is the evidence it provides that, of the excess deaths revealed in daily studies as due to cold, most, possibly all, have been displaced by at least 6 months. This in turn strengthens evidence that policies aimed at reducing vulnerability to cold (eg, home insulation) are importantly beneficial to health and specifically life expectancy.
